# Inflammatory Mechanisms of Organ Crosstalk during Ischemic Acute Kidney Injury

**DOI:** 10.4061/2012/505197

**Published:** 2011-06-09

**Authors:** Laura E. White, Heitham T. Hassoun

**Affiliations:** ^1^Department of Surgery, The Methodist Hospital and Research Institute, Houston, TX 77030, USA; ^2^The Methodist DeBakey Heart & Vascular Center, Houston, TX 77030, USA

## Abstract

Acute kidney injury (AKI) is a common complication during inpatient hospitalization, and clinical outcomes remain poor despite advancements in renal replacement therapy. AKI in the setting of multiple organ failure (MOF) remains a formidable challenge to clinicians and incurs an unacceptably high mortality rate. Kidney ischemia-reperfusion injury (IRI) incites a proinflammatory cascade and releases cellular and soluble mediators with systemic implications for remote organ injury. Evidence from preclinical models cites mechanisms of organ crosstalk during ischemic AKI including the expression of cellular adhesion molecules, lymphocyte trafficking, release of proinflammatory cytokines and chemokines, and modification of the host innate and adaptive immune response systems. In this paper, the influence of kidney IRI on systemic inflammation and distant organ injury will be examined. Recent experimental data and evolving concepts of organ crosstalk during ischemic AKI will also be discussed in detail.

## 1. Introduction

Despite advancements in renal replacement therapy, acute kidney injury (AKI) is a frequent complication with severe implications for the critically ill patient. Published mortality rates for intensive care unit patients with AKI range between 30 and 70%, and AKI alone remains an independent risk factor for mortality even after adjustment for demographics and severity of illness [1, 2]. Kidney ischemia-reperfusion injury (IRI) occurs in various clinical settings including shock, sepsis, organ transplantation, and vascular surgery. AKI, however, rarely occurs in isolation. It has become apparent that clinically much of the high patient mortality can be attributed to the onset of systemic inflammatory response syndrome (SIRS) and progression to multiple organ failure (MOF). 

Since the 1950s, clinicians have identified abnormal chest radiographs in patients with chronic kidney failure. Pulmonary dysfunction was thought to result from “increased permeability of congested pulmonary capillaries,” and this was dubbed the “uremic lung” [3]. Even in the acute setting, however, kidney dysfunction directly contributes to the onset of remote organ injury. For example, increased kidney ischemia time during complex aortic surgery is associated not only with acute and chronic renal failure, but also with an increased incidence of remote organ injury and death [4–6]. 

Clinical and translational laboratory studies have demonstrated the relevance of interactions between the injured kidney and distant organs, and complex mechanisms of crosstalk between injured kidneys and remote organs such as the lungs, liver, heart, gut, brain, and hematologic system have been identified. Recent data highlights the importance of both the innate and adaptive immune response, activation of proinflammatory cascades, and an alteration of transcriptional events in remote organs during ischemic AKI. The purpose of this paper is to review emerging concepts of organ crosstalk and recent experimental data regarding the activation and systemic expression of proinflammatory pathways during ischemic AKI. For a complete list of abbreviations used in this paper, please see Table 1.

## 2. Ischemic AKI

Kidney IRI remains a major cause of AKI in both native and transplanted organs and lacks a specific treatment aside from renal replacement therapy. The local effects of kidney IRI begin in the most vulnerable regions of the organ, after which a cascade of microvascular inflammation propagates. Although 25% of cardiac output contributes to renal blood flow, only a fraction ultimately reaches the vasa recta of the renal medulla, with the majority reaching the renal cortex [7]. Therefore, even slight alterations in total renal blood flow may lead to anoxic injury in the medulla, resulting in tubular dysfunction, salt wasting, and glomerular vasoconstriction through tubuloglomerular feedback [8, 9]. 

Kidney IRI activates an inflammatory response which results in endothelial cell activation, leukocyte adhesion and entrapment, and compromised microvascular blood flow [10]. Inflammation in the postischemic kidney triggers the upregulation of leukocyte adhesion molecules, toll-like receptors, and downstream transcription factors, which all contribute to disruption of the integrity of the renal vascular endothelium [11]. Adhesion molecules such as integrins and selectins along with proinflammatory cytokines propagate cellular injury not only locally in the renal tubular epithelial cells but also travel to remote organs where genomic markers of injury are upregulated and phenotypic injury occurs [12]. Unfortunately, selective inhibition of cytokines and adhesion molecules such as tumor necrosis factor-*α* (TNF-*α*) and intercellular adhesion molecule (ICAM-1) have failed to demonstrate global attenuation of both local and remote organ injury during experimental models of ischemic AKI [13–16]. However, *α*-melanocyte-stimulating hormone (*α*-MSH), a cytokine with broad antiinflammatory, anticytotoxic, and antiapoptotic properties, has demonstrated success in treating the inflammatory phenotype in rodents. In this model, treatment with *α*-MSH has attenuated both renal and pulmonary injury during ischemic AKI [13, 17].

**Figure 1:**
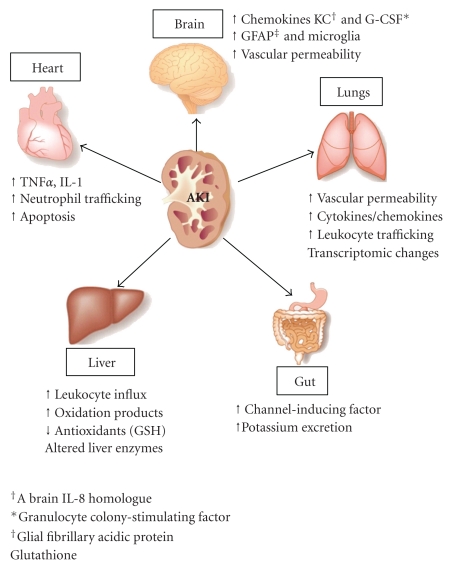
AKI and organ crosstalk. AKI induces remote organ injury in the heart, brain, lungs, liver, and gut involving multiple inflammatory pathways, including increased expression of soluble proinflammatory mediators, innate and adaptive immunity, cellular apoptosis, physiologic derangements and genomic changes.

The largely unsuccessful effort to ameliorate MOF with specific anti-inflammatory therapeutics highlights the complexity of the systemic response to kidney IRI. From these early experimental studies, it is possible that the multiple inflammatory pathways activated in each organ system represent a unique response to ischemic AKI. Despite a paucity of data, several different pathophysiologic responses to ischemic AKI in remote organs have been identified (Figure 1). In the heart, increased expression of TNF*α* and interleukin-1 (IL-1) are associated with myocyte apoptosis. In the brain, increased expression of chemokines including keratinocyte chemoattractant (KC, a brain IL-8 homologue) and granulocyte colony-stimulating factor (G-CSF) are seen with increased vascular permeability. Additional responses in the lungs, gut, and liver have been discovered, and these individual organ responses will be detailed later in this paper. We will now focus on cellular mediators, specifically those involved in the innate and adaptive immune system, which may connect local ischemic AKI with distant organ injury.

### 2.1. Innate Immunity

The innate immune system plays an important role in mediating the inflammatory response during ischemic AKI. Traditionally, innate immunity elicits an immediate, preprogrammed response to tissue injury that lacks immunologic memory. It is composed of plasma proteins (complement), cells (neutrophils, macrophages, and natural killer cells), and physical barriers. Proposed initiators of the innate immune response during IRI include the activation of toll-like receptors (TLRs) and the release of reactive oxygen species (nitric oxide and superoxide anion) and mitochondrial products [18]. The complement system, particularly the alternative pathway, is activated, stimulating release of cytokines and subsequent activation of neutrophils, endothelium, and macrophages [19]. 

TLRs are a family of transmembrane proteins which serve as major pattern recognition receptors, binding to a wide range of microbial products and endogenous ligands released as a consequence of injury. TLR-dependent signaling serves as a rapid response mechanism to local tissue damage and has been implicated in early activation of the innate immune response during kidney IRI [11, 20]. Renal tubular epithelial cells constitutively express TLR2 and TLR4, and IRI results in selective upregulation of these TLRs. Experimental studies of kidney IRI have demonstrated attenuation of renal structural and functional injury in both TLR2−/− and TLR4−/−mice, implicating TLR2 and TLR4 signaling in renal damage [21, 22]. TLR2 may also play a role in transplantation tolerance by decreasing the infiltration of T cells, dendritic cells, and macrophages [23]. 

T cells may act during early IRI despite lacking alloantigenic stimulation, which challenges the traditional function of T cells as exclusive mediators of the adaptive immune response [19]. Significant evidence exists to support the role of T cells in gut, heart, and lung ischemia-reperfusion injury [24–28]. Likewise, experimental data also points toward antigen-independent T-cell activation in ischemic AKI through cytokines secreted by macrophages and dendritic cells, chemokines, oxygen free radicals and the complement system [7, 19, 29]. 

Specific populations of T cells likely contribute to injury in unique ways. For example, mice deficient in CD4+ and CD8+ cells and athymic mice demonstrate protection from experimental ischemic AKI; however, only adoptive transfer of T cells of the CD4+ type restores this injury [30, 31]. Not all T cells, however, perpetuate inflammatory damage during ischemic AKI; some populations of T cells confer renal protection after IRI, particularly the Th_2_ phenotype of CD4+ T cells and T regulatory cells [32]. Additional recent experimental data has characterized specific populations of T lymphocytes trafficking to remote organs that may facilitate organ crosstalk during ischemic AKI. In a rodent model of kidney IRI, a distinct influx of CD3+ (T) cells with a predominant CD8+ T cell subpopulation was identified 24 hours after experimental kidney IRI. These pulmonary T cells acquired increased expression of activation markers suggesting that ischemic AKI induced trafficking of activated T cells which contributed to pulmonary injury, specifically apoptosis [33]. The specific role of subpopulations of T cells in the pathogenesis of ischemic AKI remains a stimulating topic of current and future investigation, both in the innate and adaptive immune response to kidney IRI.

### 2.2. Adaptive Immunity

The adaptive immune system relies on the specificity of antigen receptors on both B and T cells that respond to millions of various antigenic molecular structures. Once stimulated by an antigen, B cells (humoral immunity) produce specific antibodies, perform opsonization to facilitate phagocytosis, and activate the complement system. The T-cell receptor (TCR) responds to presented antigen peptides by activating macrophages to kill phagocytosed microbes, directly destroying infected cells and by releasing cytokines to promote further response (cell-mediated immunity).

Antigen-dependent T-cell activation has been demonstrated in experimental models of renal IRI [34, 35]. For example, mice with a restricted TCR cell repertoire suffer less injury and promote a far diminished inflammatory response during renal IRI. Additionally, athymic mice that subsequently underwent adoptive transfer with these TCR-restricted T cells failed to restore injury as severely as seen with transfer of wild-type T-cells [36]. T cells also play a role in long term renal modification, as an increased number of both activated and effector-memory T cells have been observed in postischemic kidneys as long as 6 weeks after IRI [37]. These T cells may in fact recognize antigens released during kidney IRI and subsequently target the kidney in an autoimmune response, leading to long-term progression of renal dysfunction. This hypothesis was demonstrated in a murine adoptive transfer model in which naïve mice received T cells from mice who were 6 weeks after kidney IRI and subsequently developed albuminuria [38]. Little is known about the role of B cells in renal IRI; however, B-cell-deficient mice demonstrated renal protection in the early phase of experimental IRI [39]. 

In summary, both the innate and adaptive immune responses play critical roles in the pathophysiology of ischemic AKI. Either following antigen stimulation or in the presence of proinflammatory chemokines and oxygen free radicals, T cells undergo early activation and serve as a bridge between adaptive and innate immunity. This specific host immune response to kidney IRI facilitates distant organ crosstalk along with soluble proinflammatory mediators and will be further discussed in the following paragraphs.

## 3. Evolving Concepts of Organ Crosstalk during Ischemic AKI

### 3.1. Kidney-Lung Crosstalk

The combination of AKI with acute lung injury (ALI) remains a formidable challenge to clinicians caring for critically ill patients. In the setting of MOF, AKI and ALI occur more frequently together than any other combination of organ systems, and predicted mortality approaches 80% [40, 41]. This exceedingly high mortality cannot be attributed solely to volume overload; leukocyte trafficking, uremic toxins, and oxidative stress mechanisms all likely contribute to this devastating clinical syndrome. For the purposes of this paper, ALI represents a P_a_O_2_/FiO_2_ ratio <300 combined with chest radiograph findings of acute bilateral infiltrates in the absence of elevated cardiac filling pressures, as defined by the America-European Consensus Conference on Acute Respiratory Distress Syndrome (ARDS) [42].

**Figure 2:**
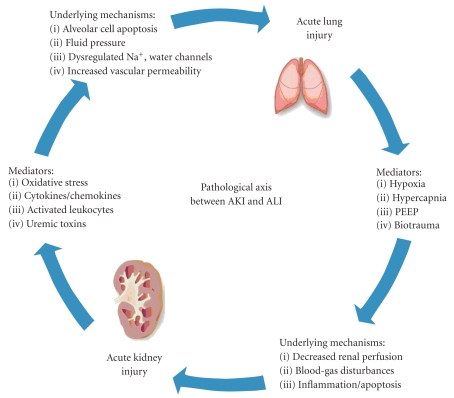
Pathological Axis between AKI and ALI. AKI induces pathophysiologic effects on the lung via cellular and soluble mediators. ALI, in turn, exacerbates kidney dysfunction through metabolic and biochemical derangements.

While the mechanisms for ischemic AKI-induced ALI remain incompletely understood, several studies point toward a self-propagating cycle with activation of proinflammatory and proapoptotic pathways (Figure 2). AKI leads to lung injury and inflammation, and in turn, ALI and its attendant hypoxemia and hypercapnia worsened by mechanical ventilation with high positive end-expiratory pressure leads to diminishing renal hemodynamics and function. Lung injury during ischemic AKI features marked pulmonary vascular permeability, erythrocyte sludging in lung capillaries, interstitial edema, focal alveolar hemorrhage, and inflammatory cell infiltration [12, 43, 44]. Mechanisms for decreased alveolar fluid clearance include downregulation of pulmonary epithelial salt and water transporters including ENaC, Na, K-ATPase, and aquaporins during ischemic AKI [43–45]. This specific response likely contributes to the increased microvascular permeability and clinical pulmonary edema frequently encountered in the setting of ischemic AKI and MOF.

#### 3.1.1. Role of Cytokines, Chemokines, and Leukocyte Trafficking

Experimental studies have identified a distinct pulmonary functional response and genomic signature induced during ischemic AKI which differs from that induced by uremia alone. A comprehensive genomic map and ontology analysis revealed many of these top differentially expressed genes participating in proinflammatory and proapoptotic pathways. Genes with early and sustained activation at 6 and 36 hours after ischemic AKI included neutrophilic granule protein (Ngp), serum amyloid A3 (Saa3), and Interleukin 1-receptor type II (Il-1r2) [12]. 

Further investigation by Deng et al. [13] has also identified an early pulmonary inflammatory response with rapid activation of transcription factors NF-*κ*B and AP-1 during ischemic AKI. By 4 hours, lung expression of TNF-*α* and ICAM-1 led to accumulation of pulmonary neutrophils. Leukocytes play a fundamental role in the development of ALI, and several studies have documented lung leukocyte activation and trafficking during experimental AKI with early and sustained neutrophil sequestration [46, 47]. Additional experiments by Klein et al. have demonstrated early expression of the neutrophil chemokine keratinocyte-derived chemokine (KC/CXCL1) and macrophage inflammatory protein (MIP-2/CXCL-2) along with increased pulmonary myeloperoxidase activity and pulmonary microvascular permeability during ischemic AKI [45]. The authors also examined interleukin-6, a neutrophil-recruiting chemokine previously identified as a candidate gene by preferential expression in whole lung tissue during ischemic AKI [48], and determined it was critical for influencing lymphocyte trafficking and pulmonary permeability [45]. 

While neutrophils are key mediators in several extrapulmonary models of ALI such as sepsis and mesenteric IRI, their role in ischemic AKI-induced ALI remains to be elucidated. Uremic neutrophils have even demonstrated a protective effect in the setting of ALI [49]. Clearly, leukocyte trafficking and the innate immune response play a complex and important role in mediating the pulmonary inflammatory response and dysfunction during renal IRI.

#### 3.1.2. Lung Apoptosis

Though not traditionally associated with a proinflammatory response, pulmonary apoptosis plays a critical role in the genomic and phenotypic response to ischemic AKI. Apoptosis of pulmonary epithelial and endothelial cells along with delayed leukocyte apoptosis occurs during ALI [50–52]. Pulmonary apoptosis disrupts tethering forces involved in cell-to-cell and cell-to-extracellular matrix interactions, leading to a loss of endothelial barrier function and increased vascular permeability [53]. In a rodent model, our laboratory has characterized caspase-dependent pulmonary apoptosis which is abrogated by the administration of a broad-spectrum caspase inhibitor. Not only did caspase inhibition attenuate the pulmonary functional injury, but the proapoptotic response to ischemic AKI appears to be mediated via a TNF receptor-1 (TNF-R-1) dependent pathway [54]. 

Our research has also demonstrated T-cell-dependent pulmonary apoptosis during ischemic AKI. Along with identification of activated T cells trafficking to the lungs during ischemic AKI, we have found that pulmonary apoptosis was subsequently attenuated in T-cell-deficient animals [33]. Further pursuit of the specific pathway for T cell and TNFR1-dependent pulmonary apoptosis remains an active focus of future investigation in our research laboratory.

### 3.2. Kidney Crosstalk with Other Distant Organs

Cardiovascular collapse is one of the most common causes of death in the setting of AKI, yet the mechanisms involved are not entirely understood [55]. Kelly has demonstrated left ventricular dilation, increased left ventricular end diastolic and end systolic diameter, increased relaxation time, and decreased fractional shortening in an experimental model of ischemic AKI [56]. Mechanisms of cardiac injury during ischemic AKI include cardiac myocyte apoptosis and neutrophil infiltration, which have been attributed to increased cardiac and systemic TNF*α*, IL-1, and ICAM-1 expression [56, 57]. When kidney ischemic time is decreased, cardiac IL-1 and ICAM expression, along with myocyte necrosis, decreased correspondingly [56].

Effects of ischemic AKI on the central nervous system (CNS) are evident clinically when mental status changes develop. Of note, renal replacement therapy fails to fully correct this CNS manifestation of renal failure [58]. Uremic toxins certainly contribute to many symptoms of encephalopathy; however, cellular and soluble inflammatory mediators have also been implicated. Preclinical data identified an increase of KC, G-CSF, and glial fibrillary acidic protein (GFAP) in the cerebral cortex and hippocampus of the brain, which may serve to recruit neutrophils to sites of neuronal damage [59]. This may result from either increased local neuronal production or an alteration of the blood-brain barrier; further research is required to delineate the source. A cell-mediated proinflammatory response has also been identified with activation of microglial cells (brain macrophages) during ischemic AKI [59].

Ischemic AKI is also implicated in oxidative stress, inflammation, apoptosis, and tissue damage in hepatocytes. Hepatic stellate cells (HSCs) regulate leukocyte trafficking and the secretion of chemokines such as IL-8, and crosstalk between HSCs likely occurs via a c-Jun N-terminal kinase pathway [60]. Oxidative stress during ischemic AKI causes hepatic malondialdehyde, an index of lipid peroxidation, to increase while total glutathione, an antioxidant, decreases [61]. Proinflammatory cytokine TNF*α* expression and hepatic cellular apoptosis is also evident during ischemic AKI [61]. 

Previous investigators and clinicians have labeled the gut as the “motor” of MOF because of its ability to amplify the systemic SIRS response in the setting of shock and gut hypoperfusion [62–64]. These mechanisms include increased intestinal permeability, interactions between host and bacterial pathogens, and propagation of toxins to distant organs via the lymphatic system [63, 65] and could potentially play a role during ischemic AKI. Clinical studies have long demonstrated the increased secretion of potassium by the colon and rectum in response to AKI [66], and recent literature linked channel-inducing factor, a potassium channel regulator found in both the kidney and colon, to ischemic AKI [67, 68]. This may provide explanation for why hyperkalemia does not universally occur during AKI. The role of the gut in response to organ crosstalk during ischemic AKI remains a fascinating topic of future investigation.

## 4. Conclusion

AKI is a frequent complication amongst hospitalized patients, with grave implications in the setting of MOF. Ischemic AKI initiates a cascade of proinflammatory pathways, and through the release of soluble mediators and activation of the host innate and adaptive immune systems, it facilitates organ crosstalk and remote organ injury. As our understanding of the postischemic kidney's role in mediating organ crosstalk continues to evolve, further laboratory investigation into the remote organ response to this devastating injury may reveal potential future therapeutic targets.

## Figures and Tables

**Table 1:** Abbreviations.

*α*-MSH	*α*-Melanocyte-stimulating hormone
AKI	Acute kidney injury
ALI	Acute lung injury
AP-1	Activator protein-1
ARDS	Acute respiratory distress syndrome
CNS	Central nervous system
G-CSF	Granulocyte colony-stimulating factor
GFAP	Glial fibrillary acidic protein
HSC	Hepatic stellate cell
ICAM-1	Intercellular adhesion molecule-1
IL-1	Interleukin-1
Il1r2	Interleukin-1 receptor type II
IRI	Ischemia-reperfusion injury
KC	Keratinocyte chemoattractant
KC/CXCL1	Keratinocyte-derived chemokine
MIP-2/CXCL-2	Macrophage inflammatory protein
MOF	Multiple organ failure
NF-*κ*B	Nuclear factor-*κ*B
Ngp	Neutrophilic granule protein
Saa3	Serum amyloid A3
SIRS	Systemic inflammatory response syndrome
TCR	T-cell receptor
TLR	Toll-like receptor
TNFR1	Tumor necrosis factor receptor-1
TNF*α*	Tumor necrosis factor-*α*
